# Prenatal Cannabis and Tobacco Co-Exposure and Its Association with Behavioural Outcomes in Middle Childhood: Co-exposition prénatale au cannabis et au tabac et son association avec les résultats comportementaux au cours de l'enfance intermédiaire

**DOI:** 10.1177/07067437241271696

**Published:** 2024-08-14

**Authors:** Emma Nadler, Joanna Jacobus, Rachel A. Rabin

**Affiliations:** 1 5620 McGill University, Montreal, Canada; 2 Department of Psychiatry, 8784 University of California San Diego, San Diego, California, USA; 3 Department of Psychiatry, McGill University and The Douglas Mental Health University Institute, Montreal, Canada

**Keywords:** cannabis, tobacco, prenatal, internalizing, externalizing, childhood, cannabis, tabac, prénatale, internaliser, externaliser, enfance, cannabis, tabac, prénatale, internaliser, externaliser, enfance

## Abstract

**Objectives:**

Cannabis legalization has triggered an increase in prenatal cannabis use. Given that tobacco is commonly co-used with cannabis, determining outcomes associated with prenatal cannabis and tobacco co-exposure is crucial. While literature exists regarding the individual effects of prenatal cannabis and tobacco exposure on childhood behaviour, there is a gap regarding their combined use, which may have interactive effects. Therefore, we investigated whether prenatal cannabis and tobacco co-exposure was associated with greater externalizing and internalizing problems in middle childhood compared to prenatal exposure to either substance alone or no exposure.

**Methods:**

Baseline data from the Adolescent Brain Cognitive Development (ABCD) Study (collected in children ages 9–11) were used to explore differences in externalizing and internalizing scores derived from the Childhood Behavior Checklist across four groups: children with prenatal cannabis and tobacco co-exposure (CT, *n* = 290), children with prenatal cannabis-only exposure (CAN, *n* = 225), children with prenatal tobacco-only exposure (TOB, *n* = 966), and unexposed children (CTL, *n* = 8,311). We also examined if the daily quantity of tobacco exposure modulated the effect of cannabis exposure on outcomes.

**Results:**

Adjusting for covariates, a 2 × 2 ANCOVA revealed significant main effects for prenatal cannabis (*p* = 0.03) and tobacco exposure (*p* < 0.001), and a significant interaction effect on externalizing scores (*p* = 0.032); no significant main effects or interactions were found for internalizing scores. However, interactions between daily quantity of cannabis and tobacco exposure significantly predicted both externalizing and internalizing scores (*p* < 0.01).

**Conclusions:**

These findings indicate that co-exposure is associated with greater externalizing problems than exposure to either substance alone, which did not differ from each other. Further, greater tobacco exposure may amplify the negative effect of cannabis exposure on both externalizing and internalizing behaviours in children. These findings underscore the need for interventions that target cannabis and tobacco co-use in pregnant women to circumvent their adverse impact on middle childhood behaviour.

**Plain Language Summary Title:**

Prenatal Cannabis and Tobacco Co-exposure and its Association with Middle Childhood Behaviours

## Plain Language Summary

Given the high rates of both cannabis and tobacco use during pregnancy, we explored if their combined use was associated with greater problematic behaviour in 10-year-old children compared to either substance alone or no substance use. We found that children with prenatal co-exposure had greater externalizing behaviours, such as attention problems and aggression, compared to children with prenatal exposure to one of the substances or no exposure. Prenatal co-exposure, cannabis-only exposure and tobacco-only exposure had no effect on childhood internalizing behaviours (e.g., depression, anxiety). However, the amount of tobacco consumed by the mother amplified the negative effect of cannabis on both childhood externalizing and internalizing behaviours. These findings emphasize the need for specialized treatment for cannabis and tobacco co-use in pregnant women to circumvent the adverse impact of these substances on externalizing behaviours in middle childhood.

## Graphical abstract

**Graphical Abstract:**
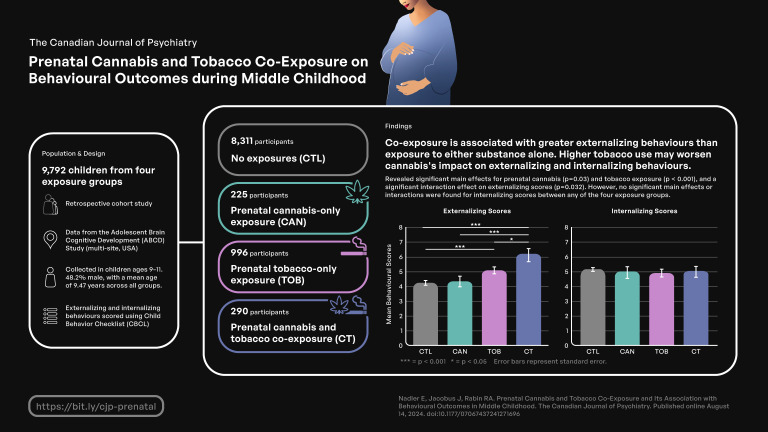
This visual abstract was created by Michael Wong.

## Résumé

**Objectifs:**

La légalisation du cannabis a provoqué une augmentation de l’usage prénatal du cannabis. Étant donné que le tabac est communément co-utilisé avec le cannabis, il est crucial de déterminer les résultats associés de la co-exposition prénatale au cannabis et au tabac. Bien que la littérature existe sur les effets individuels de l’exposition prénatale au cannabis et au tabac sur le comportement de l’enfance, il existe une lacune concernant leur utilisation combinée, qui peut avoir des effets interactifs. Par conséquent, nous avons étudié si la co-exposition prénatale au cannabis et au tabac était associée à de plus grands problèmes d'extériorisation et d'intériorisation au cours de l'enfance par rapport à l'exposition prénatale à l'une ou l'autre substance seule et à l'absence d'exposition.

**Méthodes:**

Les données de base de l’étude ABCD (Adolescent Brain Cognitive Development) (recueillies chez les enfants âgés de 9 à 11 ans) ont été utilisées pour explorer les différences dans les scores d'extériorisation et d'intériorisation dérivés de la Childhood Behaviour Checklist dans quatre groupes : les enfants ayant été co-exposés de façon prénatale au cannabis et au tabac (CT, *n* = 290), les enfants ayant été exposés de façon prénatale uniquement au cannabis (CAN, *n* = 225). les enfants ayant été exposés uniquement au tabac avant la naissance (TOB, *n* = 966) et les enfants non exposés (CTL, *n* = 8 311). Nous avons également examiné si la quantité quotidienne d'exposition au tabac modulait l'effet de l'exposition au cannabis sur les résultats.

**Résultats:**

En tenant compte des covariables, une ANCOVA 2 × 2 a révélé des effets principaux significatifs pour l'exposition prénatale au cannabis (*p* = 0,03) et au tabac (*p* < 0,001), ainsi qu'un effet d'interaction significatif sur les scores d'externalisation (*p* = 0,032) ; aucun effet principal ou interaction significatif *n*'a été trouvé pour les scores d'intériorisation. Cependant, les interactions entre la quantité quotidienne de cannabis et l’exposition au tabac prédisaient de manière significative les scores d’externalisation et d’intériorisation (*p* < 0,01).

**Conclusions:**

Ces résultats indiquent que la co-exposition est associée à des problèmes d'extériorisation plus importants que l'exposition à l'une ou l'autre substance seule, qui ne diffèrent pas les unes des autres. De plus, une plus grande exposition au tabac peut amplifier l’effet négatif de l’exposition au cannabis sur les comportements d’extériorisation et d’intériorisation chez les enfants. Ces résultats soulignent la nécessité d'interventions ciblant la consommation conjointe de cannabis et de tabac chez les femmes enceintes afin d'éviter leur impact négatif sur le comportement de l'enfance intermédiaire.

## Introduction

Cannabis legalization has led to an increase in prenatal cannabis use, likely reflecting increased availability and social acceptance.^1,2^ In the United States and Canada, ∼11% of pregnant women self-report past-month non-medical cannabis use, with rates trending upwards.^1,3,4^ Despite the well-documented harms of prenatal cannabis use on fetal development and neonatal outcomes, 30% of pregnant women do not perceive cannabis as harmful to their developing child,^5^ an assumption that remains unsupported.^6^ In fact, pregnant women may consume cannabis to combat nausea and other pregnancy-related symptoms.^7^ Yet, there is a growing public health concern that the adverse health consequences associated with prenatal cannabis exposure on offspring far outweigh the potential therapeutic effects for the mother.

Cannabis use is commonly accompanied by tobacco use. In the general population, ∼30% of individuals who use tobacco co-use cannabis,^8^ and even higher rates of tobacco co-use are reported in pregnant women who use cannabis (45%).^9,10^ Reasons for tobacco co-use may include enhancing the euphoric effects of cannabis, remedying cannabis-induced cognitive impairments, and attenuating cannabis withdrawal.^11^ High rates of co-use during pregnancy are concerning given that the primary psychoactive ingredients of cannabis and tobacco (tetrahydrocannabinol [THC] and nicotine, respectively) cross the placenta and accumulate in fetal plasma in high concentrations.^12^

The endocannabinoid system plays a critical role in orchestrating reproductive processes.^13-15^ Therefore, THC exposure may disturb endocannabinoid signalling and lead to negative fetal outcomes. Accordingly, prenatal cannabis exposure has been associated with increased risk of stillbirth, intrauterine growth restriction, and improper maturation of the neonatal brain.^13,16^ Additionally, prenatal cannabis exposure may result in long-term behavioural consequences, such as externalizing (e.g., social and attention problems, aggression) and internalizing (e.g., anxious, depressed) problems that only become apparent in childhood.

Studies have investigated associations between prenatal cannabis exposure and the subsequent development of problematic behaviours in children. Using data from the Adolescent Brain Cognitive Development (ABCD) study and controlling for confounding factors, Paul et al.^17^ reported that prenatal cannabis exposure was associated with greater externalizing problems in middle childhood (∼10 years old) relative to unexposed children. Using the same dataset, but controlling for different covariates, Cioffredi et al.^18^ similarly reported that children with prenatal cannabis exposure had greater externalizing problems compared to children matched on prenatal tobacco and alcohol exposure and unexposed children. These findings support earlier studies in a different cohort that found that prenatal cannabis exposure increased externalizing problems at age 10,^19,20^ and partially with another study that found that prenatal cannabis exposure was associated with increased aggression in 18-month-old girls, but not boys.^21^

These studies also examined internalizing behaviours; however, the results were inconsistent. While three studies reported that prenatal cannabis exposure was not associated with internalizing symptoms in young or middle childhood,^17,20,21^ Goldschmidt et al.^19^ found that prenatal cannabis was associated with fewer internalizing problems in 10-year-old children. By contrast, other studies found positive associations between prenatal cannabis exposure and internalizing symptoms in 10-year-old children.^18,22^

Nicotine binds to nicotinic acetylcholine receptors, which are developed in the fetal brain by four weeks of gestation.^23^ Prenatal nicotine exposure interferes with neurodevelopmental processes in the fetus,^24^ resulting in aberrations in birth and perinatal health outcomes (e.g., decreased in-utero brain growth, preterm delivery, and low birth weight).^25^ Like cannabis, prenatal tobacco exposure has been associated with increased externalizing symptoms in children aged 5–18,^26-28^ and specifically among children ∼10 years old.^29,30^ Sex-specific results have also been reported such that prenatal tobacco exposure was shown to disproportionally affect externalizing behaviours in three-year-old boys compared to girls.^31,32^ Yet, many studies did not observe relationships between prenatal tobacco exposure and internalizing behaviours in children.^33-36^ The studies that did find an association^37,38^ failed to control for other prenatal substance exposure, such as alcohol, which may be driving the effect.^39^

Thus, both prenatal cannabis and prenatal tobacco exposure independently increase externalizing problems in middle childhood, with less evidence supporting a relationship between these substances and internalizing problems. Therefore, it would be critical to examine potential interactions between prenatal cannabis and prenatal tobacco exposure on childhood behaviours, as their combined exposure may result in more detrimental outcomes than each substance alone given the overlapping distribution of cannabinoid and nicotinic receptors in the brain.^40,41^

Despite cannabis and tobacco co-use being common among pregnant women, consequences associated with prenatal co-exposure on childhood behavioural outcomes remain understudied. Godleski et al.^42^ compared externalizing behaviours in 24- and 36-month-old children with prenatal co-exposure, tobacco-only exposure, and no exposure; internalizing behaviours were not examined. No group differences were observed in either age group.^42^ The authors posited that the sample examined may be too young for externalizing symptoms to emerge,^43^ and the effects of prenatal co-exposure on these behaviours may lay dormant until middle childhood.^43^ Given that no study has investigated the effects of prenatal co-exposure on externalizing and internalizing symptomatology in middle childhood, we aimed to fill that gap.

Using baseline data from the ABCD study which comprises a large and diverse population of 9–11-year-old children with negligible substance use,^44^ we explored the interactive effects of prenatal cannabis and tobacco co-exposure on externalizing and internalizing behaviours in middle childhood, relative to single substance exposure and no exposure. We hypothesized that children with prenatal co-exposure would have greater externalizing problems compared to children with prenatal cannabis-only exposure, tobacco-only exposure, and unexposed children. By contrast, we did not expect any pattern of prenatal substance exposure to be associated with internalizing behaviours. Last, given that prenatal cannabis and tobacco exposure may have sex-specific effects,^31,32,45^ we explored if child sex moderated the relationship between prenatal substance exposure and childhood behaviour.

## Methods

### Participants

Data were retrieved from the ABCD Study Annual Data Release 4.0, a longitudinal study that tracks children's neurological, behavioural, and cognitive development throughout childhood. The study was approved by a central institutional review board at the University of California, San Diego, and at local sites. All parents provided written informed consent after receiving a complete description of the study, and all children provided verbal assent.

At baseline visits, data were collected from children (*n* = 11,877) aged 9–11 and their parents. The Developmental History Questionnaire asked about the biological mother's prenatal substance use (tobacco, cannabis, caffeine, alcohol, opiates/opioids, cocaine) before and after knowledge of pregnancy. Based on these responses (and exclusionary criteria presented below), children were divided into four groups: (a) children with prenatal cannabis and tobacco co-exposure (CT, *n* = 290); (b) children with prenatal cannabis-only exposure (CAN, *n* = 225); (c) children with prenatal tobacco-only exposure (TOB, *n* = 966); (d) no prenatal cannabis or tobacco exposure (CTL, *n* = 8,311). Children were included in a substance-using group if the mother used the substance before or after knowledge of pregnancy and in the unexposed group if the mother did not use cannabis or tobacco before or after knowledge of pregnancy.

In addition to the ABCD Study exclusion criteria (https://abcdstudy.org/families/participation) which includes a child's lack of English proficiency, inability to complete an MRI at baseline, and intellectual, neurological, or mental problems which would inhibit the child's ability to comply with the protocol, participants were also excluded if (i) on the Developmental History Questionnaire the response was “I don’t know” (*n* = 455) or data was missing (*n* = 9); (ii) the Developmental History Questionnaire was not filled out by the biological mother (*n* = 1,414); (iii) while pregnant with the child, the mother reported, on average, more than seven drinks per week after knowledge of pregnancy, which is in line with other research examining prenatal cannabis/tobacco use^42^ (*n* = 7); (iv) while pregnant with the child, the mother used illicit substances before *or* after knowledge of pregnancy (*n* = 50); (v) at random, one child per family of twins (*n* = 150).

### Measures

Demographic information was collected using the ABCD Developmental History Questionnaire and the ABCD Longitudinal Parent Demographic Survey.

#### The Developmental History Questionnaire

The Developmental History Questionnaire was used to retrospectively assess the child's prenatal substance exposure. The following questions were asked to the biological mother: “Before you found out you were pregnant, but while you might have been pregnant with this child, were you using cannabis?” “Once you knew that you were pregnant, were you using cannabis?” “Before you found out you were pregnant, but while you might have been pregnant with this child, were you using tobacco?” and “Once you knew you were pregnant, were you using tobacco?” (0 = No, 1 = Yes, 999= Don’t know). This questionnaire also assessed the frequency of substance use by asking, “Once you found out you were pregnant, how many times per day did you smoke [cannabis/tobacco]?” This questionnaire also assessed maternal alcohol and illicit substance use during pregnancy using the following questions. “Once you knew that you were pregnant, were you using [alcohol, cocaine/crack, oxycontin, heroin/morphine]?” or “Before you found out you were pregnant, but while you might have been pregnant with this child, were you using [alcohol, cocaine/crack, oxycontin, heroin/morphine]?”.

#### Child Behavior Checklist (CBCL)

Our primary outcomes of interest were derived from the CBCL.^46^ The CBCL is a parent-reported questionnaire that describes their child's behaviour and emotional problems. Parents answer 118 questions scored on a 3-point Likert scale (0 = absent, 1 = occurs sometimes, 2 = occurs often). Each behaviour assessed was classified into one of eight syndromes: anxious/depressed, depressed, somatic complaints, social problems, thought problems, attention problems, rule-breaking behaviour, and aggressive behaviour. Behavioural scores were added up in each category to produce a raw syndrome score. Behavioural scores were also compiled into two broader scores yielding an externalizing severity raw score and an internalizing severity raw score, with higher scores denoting greater problem severity. Externalizing scores were compiled from attention problems, delinquency, and aggressive behaviour, while internalizing scores were compiled from anxious, depressed, and somatic complaints.

#### Achenbach System of Empirically Based Assessment (ASEBA)

Parent externalizing and internalizing scores were derived from the parent self-report ASEBA. These scores were included as covariates in their respective models.

### Statistical Analysis

Analyses were conducted in SPSS Version 29.0. Between-group differences in demographic and perinatal variables were tested using Chi-square tests for categorical variables and a one-way analysis of variance (ANOVA) for continuous variables. We conducted two 2 × 2 analyses of covariance (ANCOVA) to test for the main effects of prenatal cannabis exposure and tobacco exposure and their interaction on externalizing and internalizing scores.^47^ Demographic and perinatal characteristics that showed significant between-group differences and were significantly correlated with the dependent variables (externalizing and internalizing scores) were included in their respective models as covariates. Separate models were run for externalizing and internalizing scores. Significant effects were followed up with Bonferroni-corrected post-hoc tests. Multiple linear regressions were employed to test for associations between the daily quantity of cannabis (i.e., cannabis use per day) and the daily quantity of tobacco (i.e., cigarettes per day) self-reported by the mother and externalizing and internalizing scores. We also created an interaction term between cannabis use per day and tobacco use per day to test for its association with externalizing and internalizing scores. Mean centring was employed for the interaction terms. The same covariates used in the ANCOVA models were employed in the respective regression models. In addition, we included the daily quantity of tobacco as a covariate in the daily quantity of cannabis predicting externalizing and internalizing scores, and similarly, we included the daily quantity of cannabis as a covariate in the daily quantity of tobacco predicting externalizing and internalizing scores. Given that 6 models were run, we applied a Bonferroni correction to account for multiple comparisons, (*p* < 0.05/6 = 0.008). Lastly, we explored if there were group-by-sex interactions on externalizing and internalizing scores using 2 × 2 × 2 ANCOVAs.

## Results

### Sample Characteristics

Sample characteristics are detailed in Table 1. Mothers of the CAN and CT groups used similar amounts of cannabis per day before and after knowledge of pregnancy. Likewise, mothers in the TOB and CT groups consumed comparable amounts of tobacco per day before and after knowledge of pregnancy. Notably, prenatal caffeine exposure did not differ across groups, thus it was not included as a covariate in any analysis.

**Table 1.** Sample Characteristics.

Variables	CTL (*n* = 8,311)	CAN (*n* = 225)	TOB (*n* = 966)	CT (*n* = 290)
Child variables				
Child age	9.47 (0.51)	9.50 (0.53)	9.50 (0.51)	9.45 (0.50)
Child sex, *n* (%)				
Male	3,994 (48.1)	119 (52.9)	472 (48.9)	132 (45.5)
Child gender, *n* (%)^1^				
Male	4,313 (51.9)	105 (46.7)	493 (51.9)	158 (54.5)
Female	3,985 (47.9)	119 (52.9)	471 (48.8)	130 (44.8)
Trans male	2 (0.0)	0 (0.0)	0 (0.0)	0 (0.0)
Trans female	3 (0.0)	0 (0.0)	0 (0.0)	0 (0.0)
Gender queer	0 (0.0)	0 (0.0)	1 (0.1)	0 (0.0)
Other	2 (0.0)	1 (0.4)	1 (0.1)	0 (0.0)
Refused to answer	1 (0.0)	0 (0.0)	0 (0.0)	1 (0.3)
Race, *n* (%)				
White^1^	6,308 (75.9)	125 (55.6)	695 (72.0)	162 (55.9)
Black^1^	1,561 (18.8)	99 (44.0)	291 (30.1)	140 (48.3)
Native American^1^	223 (2.7)	12 (5.3)	58 (6.0)	12 (4.1)
Alaska Native	3 (0.0)	0 (0.0)	1 (0.1)	0 (0.0)
Hawaiian Native	13 (0.2)	1 (0.4)	2 (0.2)	0 (0.0)
Guamanian	1 (0.0)	0 (0.0)	1 (0.1)	0 (0.0)
Samoan	8 (0.1)	0 (0.0)	0 (0.0)	1 (0.3)
Other Pacific Islander	27 (0.3)	2 (0.9)	1 (0.1)	1 (0.3)
Asian Indian	69 (0.8)	1 (0.4)	6 (0.6)	2 (0.7)
Chinese	139 (1.7)	1 (0.4)	5 (0.5)	0 (0.0)
Filipino	113 (1.4)	6 (2.7)	9 (0.9)	6 (2.1)
Japanese	62 (0.7)	0 (0.0)	4 (0.4)	1 (0.3)
Korean	57 (0.7)	1 (0.4)	9 (0.9)	0 (0.0)
Vietnamese	41 (0.5)	0 (0.0)	4 (0.4)	0 (0.0)
Other Asian	62 (0.7)	1 (0.4)	7 (0.7)	0 (0.0)
Other Race	544 (6.5)	15 (6.7)	58 (6.0)	19 (6.6)
Maternal years of education^1^	16.77 (2.75)	15.98 (2.43)	15.29 (2.50)	15.03 (2.40)
Household income, *n* (%)^1^				
<$5,000	275 (3.3)	9 (4.0)	69 (7.1)	25 (8.6)
$5,000–11,999	250 (3.0)	17 (7.6)	68 (7.0)	37 (12.8)
$12,000–15,999	166 (2.0)	16 (7.1)	34 (3.5)	19 (6.6)
$16,000–24,999	324 (3.9)	19 (8.4)	61 (6.3)	26 (9.0)
$25,000–34,999	410 (4.9)	27 (12.0)	96 (9.9)	28 (9.7)
$35,000–49,999	607 (7.3)	18 (8.0)	109 (11.3)	33 (11.4)
$50,000–74,999	1,009 (12.1)	29 (12.9)	147 (15.2)	32 (11.0)
$75,000–99,999	1,122 (13.5)	22 (9.8)	112 (11.6)	26 (9.0)
$100,000–199,999	2,493 (30.0)	34 (15.1)	131 (13.6)	25 (8.6)
>$200,000	945 (11.4)	15 (6.7)	24 (2.5)	5 (1.7)
don't know	367 (4.4)	7 (3.1)	52 (5.4)	9 (3.1)
refuse to answer	343 (4.1)	12 (5.3)	63 (6.5)	25 (8.6)
Perinatal variables				
Birth weight (lbs)^1 2^	6.64 (6.05)	6.71 (1.414)	6.42 (1.43)	6.43 (1.42)
Birth complications, *n* (%)	2,266 (27.3)	61 (27.1)	293 (30.3)	90 (31.0)
Unplanned pregnancy, *n* (%)^1 4^	2,783 (33.5)	155 (68.9)	602 (62.2)	213 (73.4)
Maternal age at birth^1 3^	29.87 (6.05)	26.36 (6.17)	26.99 (5.67)	24.48 (5.47)
Substance use variables				
Before knowledge of pregnancy, alcohol use, *n* (%)^1^	1,624 (19.5)	118 (52.4)	388 (40.2)	177 (61.0)
Before knowledge of pregnancy, drinks per week^1^	0.58 (1.85)	2.22 (4.58)	1.76 (3.62)	2.88 (4.57)
After knowledge of pregnancy, alcohol use, *n* (%)^1^	143 (1.7)	16 (7.1)	21 (2.2)	10 (3.4)
After knowledge of pregnancy, drinks per week^1^	0.02 (0.18)	0.12 (0.71)	0.04 (0.33)	0.03 (0.29)
During pregnancy caffeine drinks per week	0.55 (1.9)	0.47 (1.27)	0.54 (1.56)	0.59 (1.74)
Before knowledge of pregnancy cannabis use, *n* (%)	0 (0.0)	222 (98.7)	0 (0.0)	288 (99.3)
Before knowledge of pregnancy, cannabis use per day^5^	0 (0.0)	1.45 (1.34)	0 (0.0)	1.86 (3.11)
After knowledge of pregnancy cannabis use, *n* (%)	0 (0.0)	62 (27.6)	0 (0.0)	90 (31.0)
After knowledge of pregnancy, cannabis use per day^6^	0 (0.0)	0.48 (1.07)	0 (0.0)	0.45 (1.02)
Before knowledge of pregnancy tobacco use, *n* (%)	0 (0.0)	0 (0.0)	962 (99.6)	287 (99.0)
Before knowledge of pregnancy, tobacco use per day^7^	0 (0.0)	0 (0.0)	7.61 (6.54)	7.15 (6.23)
After knowledge of pregnancy tobacco use, *n* (%)	0 (0.0)	0 (0.0)	322 (33.3)	103 (35.5)
After knowledge of pregnancy, tobacco use per day^8^	0 (0.0)	0 (0.0)	2.24 (4.60)	1.95 (3.84)
Parent behaviour variables				
Parent externalizing^1,9^	4.87 (5.0)	8.51 (6.7)	7.95 (6.7)	11.53 (8.5)
Parent internalizing^1,9^	8.92 (8.1)	12.48 (10.0)	13.06 (10.5)	17.00 (12.1)
Child behaviour variables				
Child externalizing^1,10^	3.97 (5.3)	5.66 (6.7)	6.20 (7.2)	8.88 (8.6)
Child internalizing^1,10^	4.88 (5.4)	6.02 (5.5)	6.13 (6.2)	7.50 (7.2)

^1^ Significant between-group differences (*p* < 0.05); ^2^Missing data (*n* = 160), ^3^Missing data (*n* = 90), ^4^Missing data (*n* = 1), ^5^Missinig data (*n* = 87), ^6^Missing data (*n* = 23), ^7^Missing data (*n* = 82), ^8^Missing data (*n* = 29),^ 9^Missing data (*n* = 3), ^10^Missing data (*n* = 4).

Mean and standard deviation are presented unless otherwise stated.

CAN, children with prenatal cannabis-only; CT, children with prenatal cannabis and tobacco exposure; exposure; CTL, unexposed children; *n*, number; SD, standard deviation; TOB, children with prenatal tobacco-only exposure.

The following variables differed between groups and correlated with externalizing scores and were therefore included as covariates in the externalizing model: child gender, birth weight, unplanned pregnancy, maternal age at birth, before knowledge of pregnancy alcohol use and drinks per week, maternal education level, and household income. Similarly, the following variables correlated with internalizing scores and were included as covariates in the internalizing model: birth weight, unplanned pregnancy, maternal age at birth, before knowledge of pregnancy alcohol use and drinks per week, maternal education level, and household income. The ethnoracial distribution differed across groups for White, Black, and Native American. However, these variables provide descriptors of the sample and were not employed as covariates in our analyses as to remain consistent with recent guidelines on the use of such variables in biomedical research.^48^

Of note, we found that externalizing scores were significantly lower among participants whose data were included compared to participants whose data were missing (*p* = 0.03), while internalizing scores were comparable between groups (*p* = 0.09).

### Externalizing Scores

**Figure 1.**
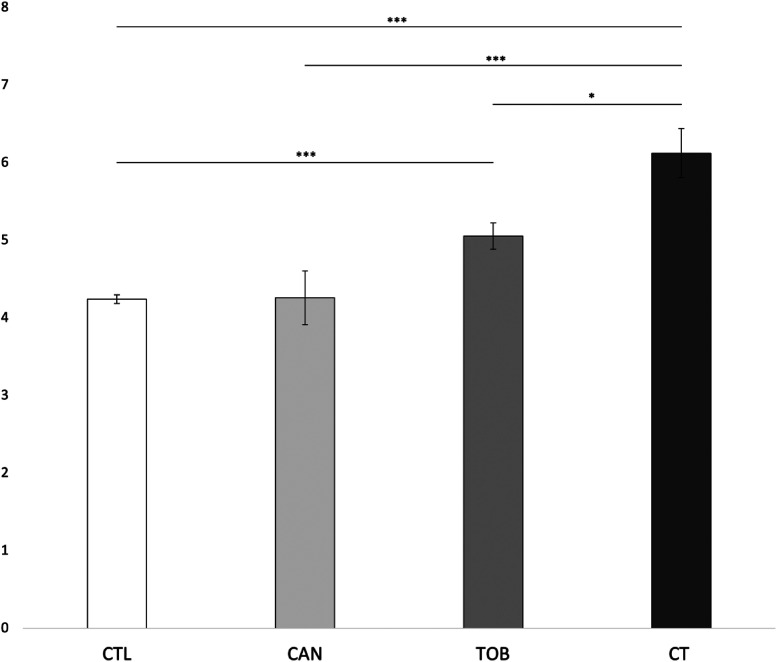
Group differences in externalizing scores. ***p < 0.001; *p < 0.05. CAN, children with prenatal cannabis-only exposure; CT children with prenatal cannabis and tobacco exposure; CTL, unexposed children; TOB, children with tobacco-only exposure; Error bars represent standard error. CT had greater externalizing scores compared to CAN, TOB, and CTL. CAN and TOB, and CAN and CTL did not differ in externalizing scores. CTL had the lowest externalizing score that differed significantly from TOB and CT.

**Figure 2.**
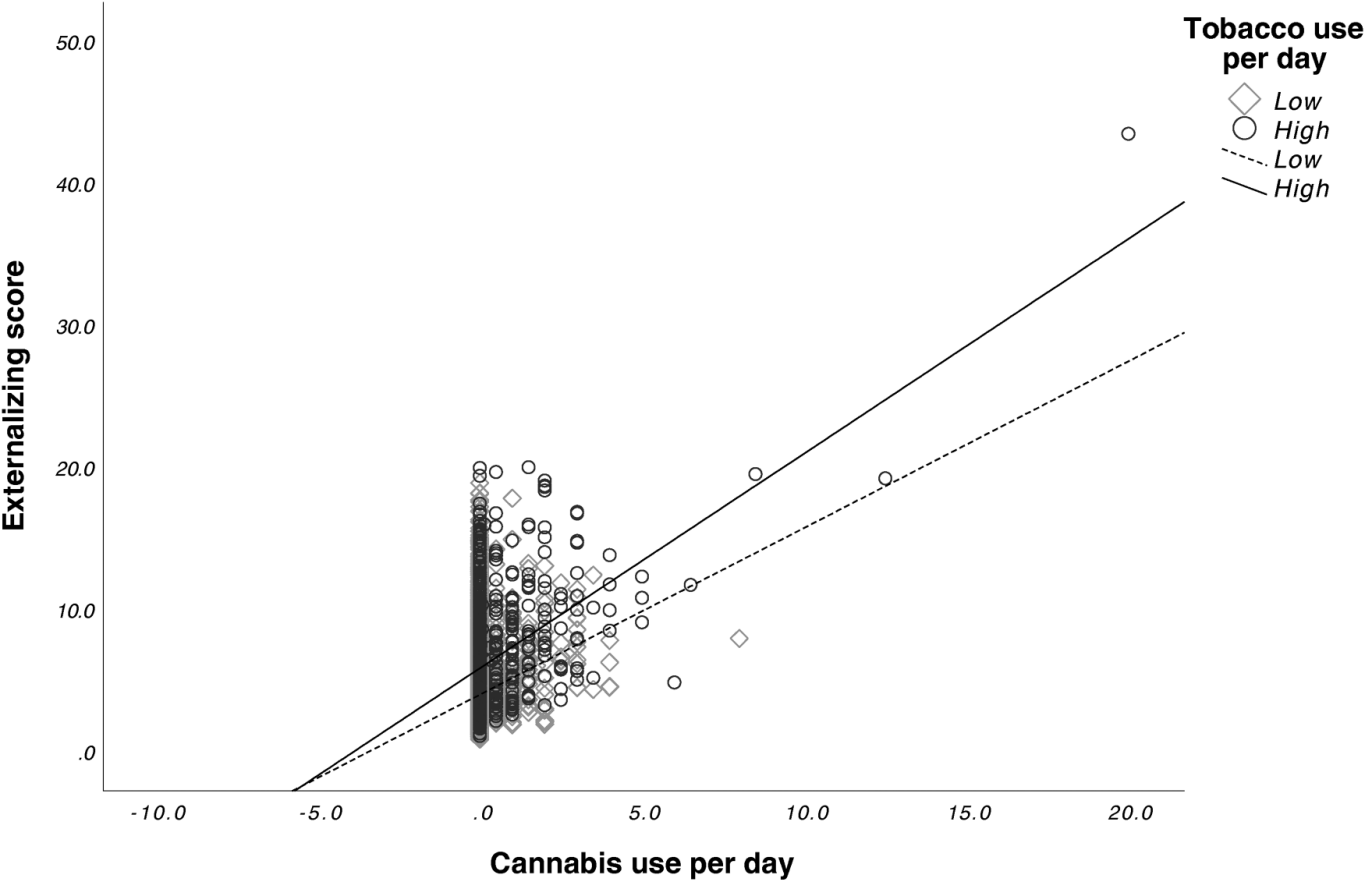
Interactive effects of prenatal cannabis exposure and tobacco exposure on externalizing scores. Greater tobacco use amplified the positive association between cannabis use frequency and externalizing behaviours, resulting in higher predicted externalizing scores as cannabis use increased. By contrast, for those with lower tobacco use, the increase in externalizing scores with higher cannabis use was less pronounced. Cannabis use per day and tobacco use per day were mean-centered. Tobacco use per day was divided into high and low groups around the mean use per day (0.61).

We found a significant main effect for prenatal cannabis exposure (*F*_1,9545 _= 4.702, *p* = 0.03) and prenatal tobacco exposure (*F*_1,9545 _= 28.959, *p* < 0.001) on externalizing scores. The interaction effect between prenatal cannabis exposure and prenatal tobacco exposure was also significant (*F*_1,9545 _= 4.615, *p* = 0.032). Bonferroni corrected post-hoc tests revealed that the CT group (*M* = 8.88, SD = 8.6) had significantly higher externalizing scores compared to the CAN group (*M* = 5.66, SD = 6.7) (*p* < 0.001), the TOB group (*M* = 6.20, SD = 7.2) (*p* = 0.013), and the CTL group (*M* = 3.97, SD = 5.3) (*p* < 0.001). The TOB group had significantly higher externalizing scores than the CTL group (*p* < 0.001). No differences were found between the CAN and CTL groups (*p* = 0.99) or between the CAN and TOB groups (*p* = 0.27). See Figure 1. Lastly, average cannabis use per day (β = 0.64 (0.13), *p* < 0.001) and average cigarettes per day (β = 0.09 (0.02), *p* < 0.001) during pregnancy were significantly associated with externalizing scores. The interaction between average cannabis use per day and average cigarettes per day on externalizing scores was also significant (β = 0.05 (0.02), *p* = 0.002). See Figure 2.

### Internalizing Scores

**Figure 3.**
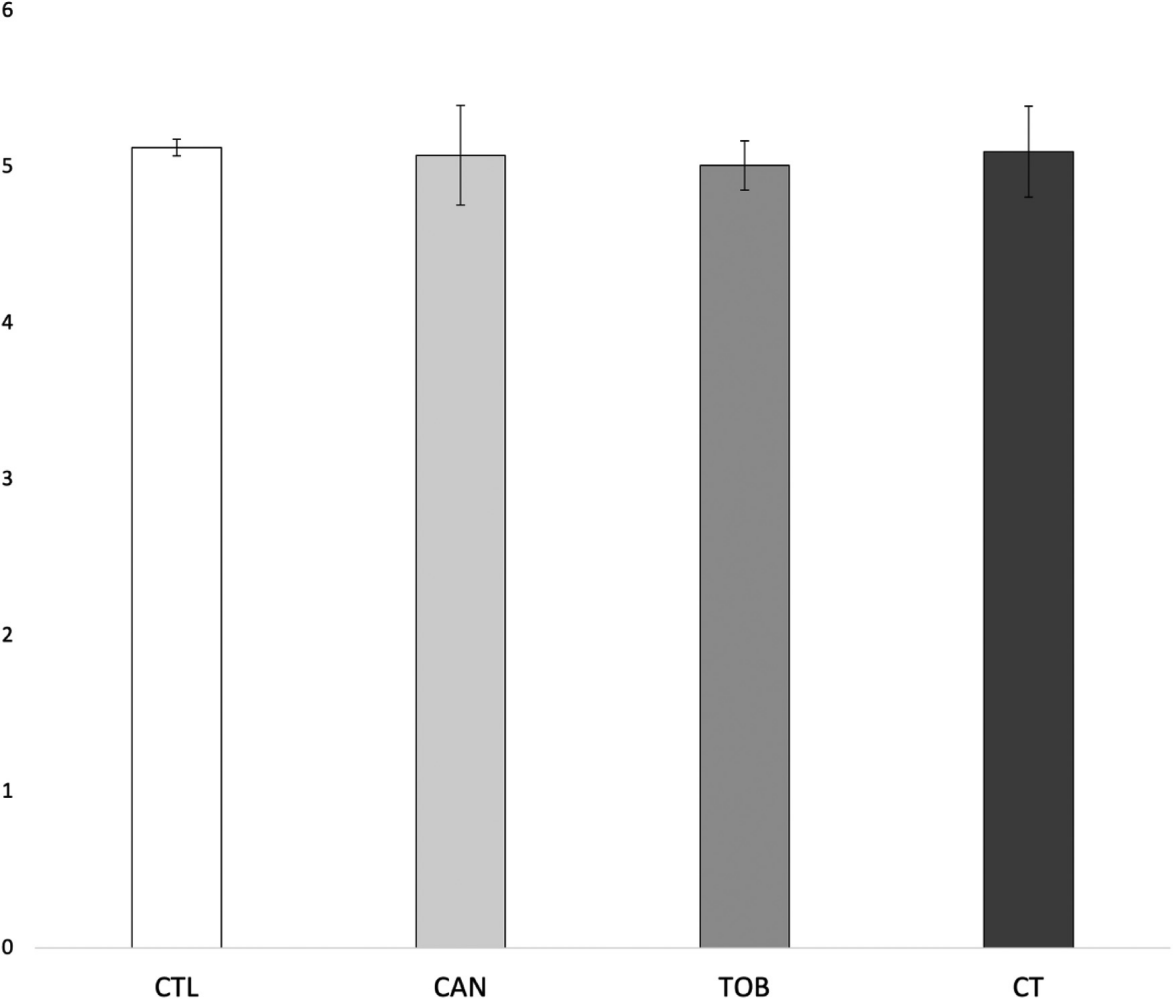
Group differences in internalizing scores. CAN, children with prenatal cannabis-only exposure; CT children with prenatal cannabis and tobacco exposure; CTL, unexposed children; TOB, children with tobacco-only exposure; Error bars represent standard error. CTL, CAN, TOB, and CT did not differ in internalizing scores.

**Figure 4.**
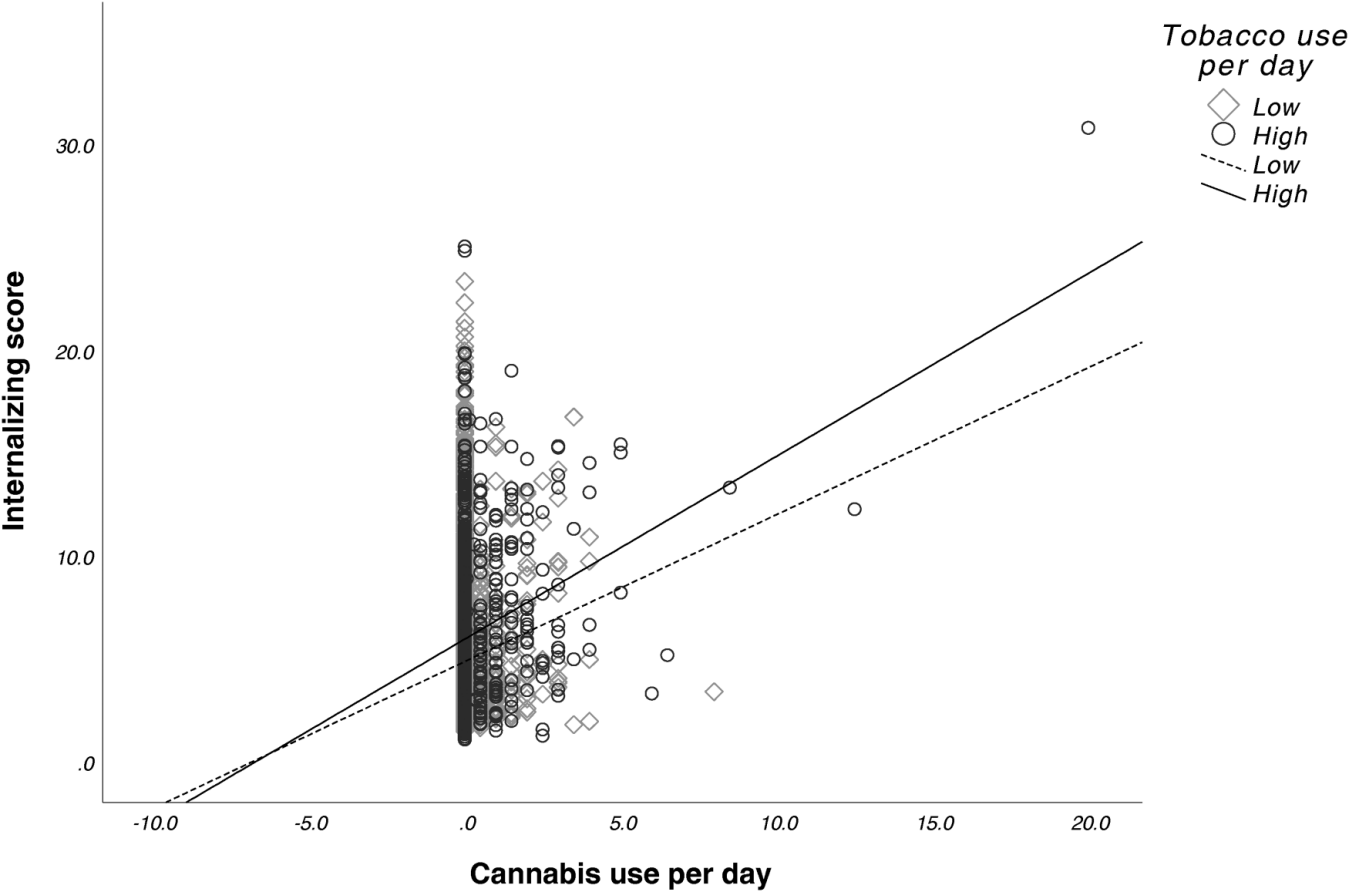
Interactive effects of prenatal cannabis exposure and tobacco exposure on internalizing scores. Greater tobacco use amplified the positive association between cannabis use frequency and internalizing behaviours, resulting in higher predicted internalizing scores as cannabis use increased. By contrast, for those with lower tobacco use, the increase in internalizing scores with higher cannabis use was less pronounced. Cannabis use per day and tobacco use per day were mean-centred. Tobacco use per day was divided into high and low groups around the mean use per day (0.61).

There were no significant main effects for prenatal cannabis exposure (*F*_1,9546 _= 0.007, *p* = 0.935) or prenatal tobacco exposure (*F*_1,9546_ = 0.040, *p* = 0.841) or their interaction (*F*_1,9546 _= 0.094, *p* = 0.760) on internalizing scores. See Figure 3. Lastly, average cannabis use per day (β = 0.16 (0.12), *p* = 0.17) and average cigarettes per day (β = -0.43 (0.02), *p* = 0.05) during pregnancy did not predict internalizing scores. However, we observed a significant interaction between average cannabis use per day and average cigarettes per day on internalizing scores (β = 0.05 (0.02), *p* < 0.001). See Figure 4.

### Group × Sex Interactions

No significant interactions between sex and group were found for externalizing (*F*_1,9541 _= 0.033, *p* = 0.855) or internalizing scores (*F*_1,9541 _= 0.212, *p* = 0.646).

## Discussion

This is the first study to investigate the interaction between prenatal cannabis and tobacco exposure on externalizing and internalizing behaviours in middle childhood. Additionally, we are the first to examine the association between prenatal cannabis exposure independent of tobacco exposure on these outcomes.

Findings from this study demonstrated both main and interaction effects of prenatal cannabis exposure and prenatal tobacco exposure on externalizing symptoms in middle childhood. We found that children with prenatal cannabis and tobacco co-exposure had greater externalizing symptoms compared to all other groups: children with prenatal cannabis-only exposure, children with prenatal tobacco-only exposure, and unexposed children. While children with prenatal tobacco exposure had greater externalizing symptoms than unexposed children, children with prenatal cannabis exposure did not differ from unexposed children. Last, there was no difference in externalizing scores between children with prenatal cannabis exposure and those with tobacco exposure.

Only one other study has examined the link between prenatal co-exposure and externalizing problems in children. Contrasting our findings, Godleski and colleagues^42^ found that prenatal co-exposure was not directly associated with externalizing behaviours in children 24 and 36-months old. Their null effect may reflect the absence of externalizing problems at this young age given that greater expression and stability of externalizing behaviours typically emerge in middle childhood.^43^

Mechanisms underlying interactions between prenatal cannabis and tobacco exposure have not yet been investigated, and thus remain unclear. Crosstalk between the endogenous cannabinoid and nicotinic systems may facilitate this effect,^49^ supported by the large overlapping distribution of cannabinoid and nicotinic receptors in the brain.^40,41^ Accordingly, prenatal cannabis exposure may sensitize the brain to tobacco's effects on neurodevelopment. In support of this, THC has been found to increase the response of the α7 nicotinic receptor to acetylcholine by 128%.^50^ Notably, this receptor subtype is over-expressed in the fetal brain and plays a critical role in neurodevelopmental processes including modulating dendritic outgrowth, establishing neural connections, and synaptogenesis.^51,52^ Likewise, tobacco exposure may potentiate cannabis’ negative effects on brain development.^53^ One possible pathway for this is via the cannabinoid 1 receptor (CB1R), given that tobacco use has been shown to affect CB1R availability.^54^ Thus, prenatal exposure to both cannabis and tobacco may generate synergistic deleterious effects on brain development which may ultimately result in greater externalizing symptomatology in middle childhood relative to either substance alone.

We also found that prenatal tobacco exposure, independent of prenatal cannabis exposure, increased externalizing problems in middle childhood compared to unexposed children, consistent with previous research.^26,27,55,56^ Prenatal tobacco exposure may lead to greater disturbances in neural maturational processes compared to prenatal cannabis exposure. For example, a previous study found that children with prenatal tobacco exposure had smaller global brain volumes compared to unexposed children, while cannabis-exposed children did not differ from unexposed children.^57^ Further, an ABCD study found that lower global brain volume was associated with greater severity of externalizing behaviours.^58^ Thus, prenatal tobacco exposure may exert a more potent influence on the developing architecture of the brain, compared to cannabis exposure, leading to more severe behavioural problems later in childhood.

Prenatal cannabis exposure, independent of tobacco exposure, showed no difference in externalizing scores compared to unexposed children, which is at odds with other studies.^17-21^ However, these samples included high proportions of children (≥50%) with prenatal tobacco exposure^17,18,20,21^ or had mothers that used on average ∼10 cigarettes per day.^19^ Thus, in these studies, the interaction between prenatal cannabis exposure and tobacco exposure may be driving the observed “cannabis” effect.

Our findings also demonstrate the importance of the level of substance exposure on externalizing outcomes. We found that greater prenatal cannabis exposure alone and prenatal tobacco exposure alone predicted higher externalizing scores. Furthermore, we observed an interaction between prenatal cannabis exposure and tobacco exposure on externalizing scores. Specifically, individuals with higher cannabis use showed a stronger positive association between tobacco use and externalizing scores compared to individuals with lower cannabis use. This suggests that greater prenatal tobacco exposure may amplify the negative effect of prenatal cannabis exposure on externalizing behaviours in middle childhood.

In line with our hypotheses, internalizing behaviours did not differ between groups. Greater prenatal cannabis exposure alone and prenatal tobacco exposure alone were not associated with internalizing scores. This was not surprising given that many studies failed to find an effect of prenatal cannabis exposure^20,21^ and tobacco exposure^33-36^ on these behavioural outcomes in children. However, we observed an interaction between prenatal cannabis exposure and tobacco exposure on internalizing scores. Thus, like externalizing behaviours, greater prenatal tobacco exposure may amplify the negative effect of prenatal cannabis exposure on internalizing behaviours in middle childhood. Accordingly, children with high prenatal cannabis and tobacco exposure may be at the greatest risk for both externalizing and internalizing problems.

Lastly, sex did not moderate the association between group and behavioural symptomatology, which may be due to the young age of the sample.^59^

This study has limitations. First, prenatal substance use data were collected retrospectively, which is subject to recall bias since mothers reported on events occurring 9–11 years prior. Second, since substance use during pregnancy is not recommended, rates of substance use may have been underreported.^60^ This may also explain why externalizing scores were significantly higher among participants whose data were missing compared to participants whose data were included in this study. Future studies should biochemically confirm the mothers’ self-reported prenatal substance use. Notably, rates of cannabis use in pregnant women from the ABCD dataset are consistent with those reported from the National Survey on Drug Use and Health,^61^ and rates of prenatal tobacco use in the ABCD dataset are consistent with rates reported by the Pregnancy Risk Assessment Monitoring System^62^ in the year the children were born. Third, substance use patterns postnatally and in the biological father were not available. Fourth, participants whose mothers consumed low levels of alcohol, defined as one drink or less per day during pregnancy, were included. The inclusion of such participants is in line with other research investigating the effects of prenatal cannabis and tobacco co-exposure,^42^ as evidence suggests that prenatal exposure at this level does not affect behavioural outcomes in the offspring.^63-65^ However, given that other studies indicate that even at low levels, prenatal alcohol exposure may alter the developmental trajectory of children,^66^ we controlled for alcohol use in our analyses. Lastly, given the increasing THC potency in cannabis over time,^67^ the cannabis used by mothers in this cohort may differ from preparations currently consumed.

## Conclusions

Results from this study contribute to the small but hopefully growing literature on the effects of prenatal cannabis and tobacco co-exposure on childhood outcomes. Our findings demonstrate the importance of both the presence and level of prenatal cannabis and tobacco exposure on externalizing and internalizing behaviours in middle childhood. Overall, this study underscores the need for interventions to address these substances among pregnant women.^68,69^
